# Gaining control: changing relations between executive control and processing speed and their relevance for mathematics achievement over course of the preschool period

**DOI:** 10.3389/fpsyg.2014.00107

**Published:** 2014-02-17

**Authors:** Caron A. C. Clark, Jennifer Mize Nelson, John Garza, Tiffany D. Sheffield, Sandra A. Wiebe, Kimberly Andrews Espy

**Affiliations:** ^1^Department of Psychology and Prevention Science Institute, University of OregonEugene, OR, USA; ^2^Developmental Cognitive Neuroscience Laboratory, Department of Psychology and Office of Research, University of Nebraska-LincolnLincoln, NE, USA; ^3^Department of Psychology, University of AlbertaEdmonton, AB, Canada; ^4^Department of Psychology, University of OregonEugene, OR, USA

**Keywords:** executive function, preschool, academic achievement, processing speed, mathematics

## Abstract

Early executive control (EC) predicts a range of academic outcomes and shows particularly strong associations with children's mathematics achievement. Nonetheless, a major challenge for EC research lies in distinguishing EC from related cognitive constructs that also are linked to achievement outcomes. Developmental cascade models suggest that children's information processing speed is a driving mechanism in cognitive development that supports gains in working memory, inhibitory control and associated cognitive abilities. Accordingly, individual differences in early executive task performance and their relation to mathematics may reflect, at least in part, underlying variation in children's processing speed. The aims of this study were to: (1) examine the degree of overlap between EC and processing speed at different preschool age points; and (2) determine whether EC uniquely predicts children's mathematics achievement after accounting for individual differences in processing speed. As part of a longitudinal, cohort-sequential study, 388 children (50% boys; 44% from low income households) completed the same battery of EC tasks at ages 3, 3.75, 4.5, and 5.25 years. Several of the tasks incorporated baseline speeded naming conditions with minimal EC demands. Multidimensional latent models were used to isolate the variance in executive task performance that did not overlap with baseline processing speed, covarying for child language proficiency. Models for separate age points showed that, while EC did not form a coherent latent factor independent of processing speed at age 3 years, it did emerge as a distinct factor by age 5.25. Although EC at age 3 showed no distinct relation with mathematics achievement independent of processing speed, EC at ages 3.75, 4.5, and 5.25 showed independent, prospective links with mathematics achievement. Findings suggest that EC and processing speed are tightly intertwined in early childhood. As EC becomes progressively decoupled from processing speed with age, it begins to take on unique, discriminative importance for children's mathematics achievement.

## Introduction

Measures of executive control (EC) have gained increasing popularity in developmental science, due in part to their strong ability to predict children's school readiness and academic achievement. For instance, children's performance on executive tasks in preschool correlates with their mathematics achievement well into elementary school (Bull et al., 2008; Clark et al., 2010; LeFevre et al., 2013). So compelling are these predictive relations that they have spurred the development of intervention programs aimed at boosting children's EC prior to school entry (e.g., Diamond et al., 2007; Bierman et al., 2008). Unfortunately, this powerful evidence for the predictive utility of executive tasks contrasts with a relatively limited understanding of the fundamental nature and development of EC as a latent construct. By definition, EC recruits and orchestrates other cognitive processes to facilitate goal-directed behavior. Measures designed to assess EC therefore are multidimensional and draw on an array of basic information processing skills, making it difficult to isolate the precise role of EC in manifest performance (Rabitt, 1997; Miyake et al., 2000; Chan et al., 2008). This conflation of EC with general information processing may be especially problematic in early childhood, when executive tasks necessarily require varied stimuli and response demands and a high degree of verbal scaffolding to promote engagement. To clearly specify the unique implications of early EC for children's academic achievement, we first need to understand how EC intersects with and diverges from basic processing abilities that also shape children's academic trajectories.

In global theories of cognitive development, processing speed is conceptualized as a central mental capacity that drives changes in higher-order cognition (Hale, 1990; Kail and Salthouse, 1994). Growth in processing speed, as assessed using simple measures of reaction time, follows a predictable, exponential pattern, independent of individual task stimuli or response requirements (Kail, 1991a,b). These age-related gains in processing speed are thought to facilitate general cognitive efficiency in two ways: (1) a greater amount of information can be absorbed within a given time frame and (2) with less time for information to decay, a larger number of neural networks can be co-activated, increasing the capacity to carry out simultaneous operations and represent information from multiple standpoints (Salthouse, 1996). Age-related changes in global processing speed therefore are argued to trigger cascading effects on higher-order systems like EC by constraining or enhancing the efficiency with which information can be processed in a domain-general manner (Kail and Salthouse, 1994; Fry and Hale, 1996).

Findings from several studies support this developmental cascade hypothesis. In literature on aging, processing speed has been found to explain an average of 75% of the variance in elderly adults' performance decline across a variety of complex cognitive tasks (Salthouse, 1996). Processing speed also accounts for between 70 and 90% of the age-related variance in fluid intelligence quotients in children and adults (Kail and Salthouse, 1994; Grudnik and Kranzler, 2001). More specific to EC, measures of processing speed have been shown to fully mediate the relation of age to inhibitory control task performance (Kail, 2002; McAuley and White, 2011) and to partially mediate the relation of age to working memory in middle childhood (Fry and Hale, 2000; McAuley and White, 2011). A seminal study by Case et al. (1982) showed that when experimental manipulations were used to equate adults and 6-year old children in their speed of information processing, their average working memory spans were equivalent. Likewise, using a latent modeling approach, where executive tasks were loaded simultaneously on EC and processing speed factors, Span et al. (2004) found that adults and school-aged children differed only in their mean processing speed and not in latent EC. Recently, Rose et al. (2011) used structural equation modeling to test the cascade model in children born preterm and full term. Consistent with a cascade effect, processing speed mediated the relation between preterm birth and impairments in EC, which in turn were associated with lower reading and mathematics achievement.

Collectively, the above studies support the idea that limitations in processing speed may constrain an individual's ability to perform more complex cognitive tasks, including the inhibition, maintenance and shifting operations attributed to EC. In fact, one conceptual model of EC includes processing speed as a key component of the executive system (Anderson, 2008; Anderson and Reidy, 2012). To date, however, no studies have examined the degree of overlap between processing speed and EC in very young children, despite the fact that increases in both processing speed and executive task performance are especially rapid during early childhood (Kail, 1991a; Wiebe et al., 2012). Given that processing speed is so ubiquitously involved in cognitive task performance, it is possible that a large proportion of the variance in young children's early executive task performance, as well as the relation of executive performance to academic achievement, may be explained by individual differences in processing speed. Addressing this question of overlap is important from a psychometric standpoint, as it challenges the very notion of EC as an independent dimension of cognition, suggesting that executive measures may not capture anything distinct from what is captured by general measures of processing speed (Salthouse et al., 2003; Fournier-Vicente et al., 2008). From a broader theoretical perspective, understanding the early relations between processing speed and EC may also yield important insights into the nature of EC development. It is conceivable, for instance, that rapid changes in myelination, synaptogenesis and connectivity during early childhood might promote system-wide changes in processing speed that facilitate executive performance in a bottom-up manner. On the other hand, temporally specific changes in frontal neural systems may promote relatively discrete age-related advancements in EC independent of gains in processing speed (Span et al., 2004). Clearly, these different developmental mechanisms would also suggest either more general or more specific strategies for early intervention.

One methodological approach that has proven powerful in understanding the underlying nature of EC at different stages of development is confirmatory factor analysis (CFA). The primary advantage of CFA is that it isolates the shared variance from several cognitive tasks that are selected a-priori to measure a given construct, thereby enhancing measurement precision and reducing error. Using CFA of executive tasks administered to school-aged children and adults, studies generally have identified 2–3 distinct but correlated factors that are conceptualized as separate components of EC and typically are labeled inhibitory control, working memory/updating and cognitive flexibility (Miyake et al., 2000; Huizinga et al., 2006; Friedman et al., 2008; Lee et al., 2013). A surprising and replicated finding from CFA studies in preschool-aged children has been the lack of differentiation of EC into distinct components (Wiebe et al., 2008, 2011; Hughes et al., 2010; Willoughby et al., 2010; Fuhs and Day, 2011). Specifically, these studies show that the overlapping variance in preschoolers' executive task performance is most parsimoniously modeled as a single, unitary factor. Collectively, these studies hint at potential changes in the underlying structure of EC over the course of childhood, although constraints on the number and types of executive tasks that can feasibly be administered to young children make it difficult to draw comparisons across different age groups. More importantly, a major limitation of any factor analytic approach is that it is not clear whether the common variance extracted from multiple tasks only reflects the construct of interest. Given that all measures of EC also tap other “bottom-up” processes and that global processing speed is thought to support performance across all higher-order cognitive tasks, it is likely that at least part of the overlap in an individual's performance on different executive tasks that is captured by his or her factor score can be attributed to the general speed with which he or she processes information. Accordingly, the first aim of this study was to use more sophisticated CFA models to parse the relative contributions of EC and processing speed to young children's executive performance. Manifest executive performance was assumed to reflect a combination of EC, processing speed and other individual differences, as well as task-specific error variance. Each executive task was loaded simultaneously onto an EC and a processing speed factor to capture relative demand on each of these constructs. Language proficiency also was statistically controlled for, given the recognized importance of language for EC development (Wolfe and Bell, 2007; Hughes et al., 2010). As argued by Salthouse et al. (2003), this type of model provides a stringent test of the divergent validity of EC because it directly pits the EC demands against the processing demands of the tasks.

We were particularly interested in whether the contributions of EC and processing speed to children's executive task performance might change over the preschool period. The rationale for this aim stems in part from our longitudinal findings on the structure of EC. At a broad, configural level, the shared variance from a repeatedly administered battery of executive tasks is best modeled as a unitary EC construct regardless of assessment point. At a more nuanced level, this EC factor does not show longitudinal metric or scalar invariance; there are changes in the way that executive tasks relate to the EC construct and in the degree of measurement error over time (Nelson et al., 2014). Cascade models suggest that growth in processing speed frees cognitive resources that then can be devoted to higher-order EC (Case et al., 1982). It is plausible, then, that relative contribution of EC capacities to executive task performance might gradually increase with age-related gains in processing speed. To examine this issue, multidimensional measurement models were fit at different preschool age points and metric invariance tests were performed to describe temporal changes in the EC and processing speed factor loadings.

The final study aim was to determine whether the processing speed demands of executive tasks might drive their relation to mathematics achievement. Strong associations between early EC and mathematics are conceptually appealing because mathematics often involves simultaneous processing and differential allocation of attention—e.g., remember the number of digits counted on one hand while you count the remaining fingers on the other. There is also substantial evidence, however, that children with poorer mathematics achievement generally are slower to process information (Bull and Johnston, 1997; Geary et al., 2012). In studies where covariate approaches have been used to isolate the contributions of EC and processing speed to mathematics achievement, executive measures have sometimes predicted mathematics achievement over and above measures of processing speed (Geary, 2011; Clark et al., 2013). Unfortunately, a covariate approach does not optimally capture the intersecting EC and processing speed demands of the executive tasks themselves. For instance, if executive task performance actually is confounded by underlying variation in processing speed, then two measures of the same construct essentially are competing in the covariate model. Using a CFA approach, van der Sluis et al. (2007) showed that the working memory updating component, but not the cognitive flexibility component of EC, was significantly associated with arithmetic achievement in school aged children after the non-executive demands of EC tasks also were modeled. Notably though, the proportion of arithmetic variance accounted for by the working memory factor was small (2.6%) relative to the proportion accounted for by the non-executive demands of EC tasks (30%), suggesting that the strong relations generally observed between executive task performance and mathematics achievement may largely be driven by the non-executive, baseline processing demands of the executive tasks. Here, we used a similar modeling approach with data from different preschool age points to determine the extent to which the processing speed and EC demands of executive tasks contributed to mathematics achievement, covarying also for language proficiency, over the course of the preschool period.

## Methods

The study included 388 preschoolers (193 boys, 195 girls; 286 Caucasian, 31 Hispanic, 20 African American, 1 Asian, 50 multi-racial) drawn from two Midwestern sites, a semi-rural area and a small city. A cohort-sequential design was used to control for practice effects associated with repeated testing; the majority of children (*n* = 228) were enrolled at age 3 years, with smaller numbers enrolled at 3.75 years (*n* = 57), 4.5 years (*n* = 55), and 5.25 years (*n* = 48) respectively. Retention rates for the earlier-recruited cohorts were high (90–100%). Children with developmental impairments (e.g., language delays, Autism) and families whose first language was not English were excluded from recruitment during a preliminary screening call. Families with lower SES were oversampled for greater diversity so that 44.1% of the study families were eligible for public medical assistance or free school lunch or had income levels below Health and Human Services poverty guidelines. Mean length of maternal education at study entry was 14.97 (*SD* = 2.37) years.

### Procedure

All procedures were approved by a university institutional review board. At the initial recruitment, researchers visited families' homes to obtain written, informed consent, to observe each child's home environment and to complete the Woodcock—Johnson III Brief Intellectual Ability Assessment (BIA; Woodcock et al., 2001a) with the child. Within a narrow 2-week window, children then visited a university-based laboratory to complete a battery of executive tasks, administered by a trained research technician. These laboratory visits were repeated every 9 months until the child was 5.25 years old. During visits, the child's primary caregiver was interviewed regarding the child's health and family background and also completed several questionnaires related to the child's wellbeing and behavior. At all assessment points, children were administered alternating forms of the Test of Early Mathematics Ability −3 (Ginsburg and Baroody, 2003). Additionally, the Applied Problems subtest from the Woodcock—Johnson III Ability Battery (Woodcock et al., 2001b) was administered at ages 3.75, 4.5, and 5.25 years. At study exit, children were re-administered the BIA.

### Measures

#### Executive control and processing speed

A broad array of measures, differing in content and response demands, was chosen to assess putative components of EC, including working memory, inhibitory control, and cognitive flexibility. A number of these executive tasks also comprised a baseline component or condition, where children were required simply to respond to colors or shapes as quickly as possible and demands on EC theoretically were minimal. Performance on many of the tasks was coded in Noldus Observer by trained undergraduate research assistants, who were blind to study hypotheses. Inter-rater reliability was computed based on 20% of the videos that were randomly selected for independent scoring or cross-coding by another research assistant.

*Nine Boxes* (adapted from Diamond et al., 1997) was selected to assess working memory. This self-ordered pointing-type task required children to search for hidden figurines in nine boxes with varying colors and lid shapes. During a 15 s delay between selections, the boxes were scrambled behind a screen. The most efficient search strategy entailed selecting only boxes that had not previously been selected. A maximum of 20 trials were administered, the task otherwise ceasing once all of the figurines had been retrieved or once the child had made 5 consecutive errors. Inter-rater reliability was 100%. The single dependent variable for this task was the child's maximum run of consecutive correct responses.

*Delayed Alternation* (Goldman et al., 1971; Espy, 1999) is a working memory task requiring the child to retrieve a food reward from one of two testing wells covered with neutrally-colored cups. When a child made a correct response, the reward was switched to the opposite well. Between trials, there was a 10 s delay, where the researcher verbally distracted the child while she hid the reward out of view. Three training trials were administered, followed by up to 16 test trials. The task was discontinued after 9 correct responses and the child was given credit for the remaining trials. Inter-rater reliability was 100%. The dependent variable for the task was the maximum length of consecutive incorrect responses subtracted from the maximum length of consecutive correct responses.

*Nebraska Barnyard* (adapted from Hughes et al., 1998) is a working memory span-type task requiring the child to remember increasing sequences of animal names. The task was programmed in Perl (Active State Software, Vancouver, BC, Canada) and administered on a touch-screen computer. During an initial training phase, children were presented with 9 colored buttons arranged in a grid-like pattern on the computer screen. Each button included a picture of an animal (e.g., green with a frog, pink with a pig) and emitted the sound the corresponding animal sound when pressed. Children were encouraged to memorize each animal's location. Thereafter, the pictures of the animals were removed, leaving only the colored buttons. Children were asked to push the buttons corresponding to progressively increasing sequences of animal names read by the examiner. Up to three trials were administered for each sequence level and children were given automatic credit for the third trial if they correctly completed the first two trials. The task ceased when the child was unable to repeat all three sequences of animal names at a given sequence length. Coding was completed in Noldus; inter-rater reliability was 96%. The dependent variable for this task was the total number of correct trials -1/3rd of a point was added for each correct one-animal sequence.

*Big-Little Stroop* (Kochanska et al., 2000) assessed processing speed and proactive inhibition and required children to name smaller shapes embedded within a larger shape. The task was administered in EPrime (Psychology Software Tools, Pittsburgh, PA, USA), with black and white line drawings used as stimuli. Of the 24 trials administered, 50% were conflict trials, where the embedded shapes were different from the larger shape and 50% were non-conflict trials, where the embedded shapes matched the larger shape. Prior to the onset of the test stimulus, a brief (750 ms) priming stimulus of the larger shape was presented. Inter-rater reliability was 90% for response times and 99% for accuracy, both of which were coded in Noldus. Dependent variables from this task included mean response times for correct non-conflict trials and mean accuracy for conflict trials.

A *Go/No-Go* task (adapted from Simpson and Riggs, 2006) provided a measure of response inhibition. During this task, children were instructed to press a button when a picture of a fish appeared on the computer screen (75%), but to refrain from pressing the button when a picture of a shark was presented (25% of trials). After each trial, children were shown a net, which appeared broken simultaneous with a buzzing sound if the child made an error of commission. Stimuli were presented in Eprime for 1500 ms, with an inter-stimulus interval of 100 ms. The dependent variable was dPrime (d'; the standardized ratio of hits to misses).

The *Modified Snack Delay* task (adapted from Kochanska et al., 1996; Korkman et al., 1998) was used to assess motor inhibition. Children were instructed to maintain a still posture and remain completely silent with their hands positioned on a mat until the researcher rang a bell after 240 s. A handful of M & M candies was positioned under a transparent glass in front of the child. At specific intervals during the delay, the researcher implemented a scripted set of distracters designed to break the child's pose (e.g., coughing, dropping a pencil, leaving the room for 1.5 min to fetch more candy). Inter-rater reliability was 90%. A hand movement score was used as the dependent variable; children were allocated a point for each epoch with no hand movement, half a point for epochs with some hand movement and 0 points for lots of hand movement. If the child ate the candy, the movement score was calculated based on the epochs completed prior to that point.

A computerized version of the *Shape School* (Espy, 1997) task provided measures of baseline processing speed, response inhibition and cognitive flexibility. Children were presented with cartoon stimuli that varied on the dimensions of color (red, blue), shape (circle, square), emotion (happy, sad), and cue (wearing a hat, not wearing a hat). For the first, baseline task condition (12 trials), children were instructed to name the colors of the characters as quickly as possible as they were presented on the computer screen. For the Inhibit condition (18 trials), children were instructed to name only characters with happy faces and to suppress naming for characters with sad faces. For the final, switching condition (15 trials), children were required to alternate their responses in accordance with a cue; characters wearing hats were to be named by their shapes and characters without hats by their color. Response times and accuracy were coded in Noldus, with inter-rater reliability being 94 and 99% for each respectively. Dependent variables were the mean reaction time for accurate baseline color naming trials, the proportion of correct inhibit trials and the proportion of correct switch trials.

*Trails-Preschool* (Espy and Cwik, 2004) was used to assess cognitive flexibility. The task was presented as a story about a family of dogs. During a baseline condition (Trails-P:A), children were asked to stamp the dogs in order of size as quickly as possible. During the subsequent, switching phase of the task (Trails-P:B), children were requested to stamp the dogs and their corresponding bones—also ordered by size—in an alternating sequence. When the child made an incorrect response, he/she was prompted to repeat the response until correct. Performance was coded in Noldus; inter-rater reliability was 99% for response times and 95% for accuracy. Dependent variables included mean reaction time for correct responses during baseline condition A and an efficiency score, computed as the correct responses/total responses for condition B.

The *Visual Matching Test* from the BIA (Woodcock et al., 2001a) was selected as a direct measure of processing speed. In the first segment of the task, children were timed as they pointed to matching shapes as quickly as possible. Following this, they were provided a pencil and asked to circle matching digits as quickly as possible within a 3 min window. Published test-retest reliability is adequate (*r* = 0.80) in the 2–7 years age range.

#### Mathematics achievement

*The Test of Early Mathematics Ability -3* (TEMA-3; Ginsburg and Baroody, 2003) was administered at each follow-up point to assesses children's rudimentary knowledge of numeric concepts, including magnitude comparison, non-verbal addition and subtraction, cardinality, part-whole relationships, mathematic symbol recognition, and counting. The TEMA-3 shows high internal (α = 0.92 − 0.96) and test-retest reliability (*r* = 0.82 − 0.93).

The *Applied Problems* subtest from the Woodcock-Johnson Tests of Achievement-III was used to assess children's early mathematical problem-solving abilities at after age 3. The task includes story and picture-based mathematical problems. Test-retest reliability in the younger age ranges is 0.92.

#### Verbal ability

The *Verbal Comprehension* subtest from the BIA (Woodcock et al., 2001a) was used as a measure of language proficiency. The subtest has four components: picture vocabulary, synonyms, antonyms, and verbal analogies. Test-retest reliability in this age range is high (*r* = 0.93).

### Analytic overview

**Figure 1:**
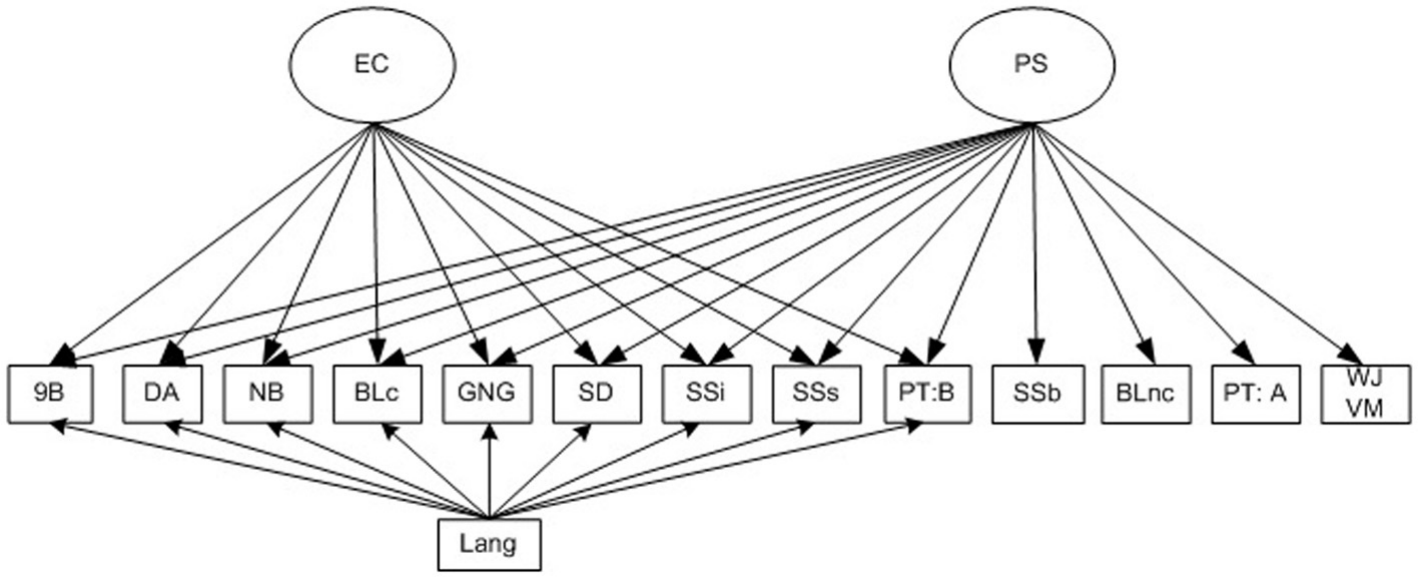
**Model of the relations between executive control and processing speed tested at different preschool age points**. EC, Executive Control; PS, Processing Speed; 9B, Nine Boxes maximum correct run; DA, Delayed Alternation score; NB, Nebraska Barnyard trials correct; BLc, Big-Little conflict trial accuracy; SD, Snack Delay movement score; SSi, Shape School Inhibit Accuracy; SSs, Shape School Switch accuracy; PT:B, Preschool Trails: B efficiency; SSb, Shape School Baseline naming response time; BLnc, Big-Little non-conflict trail response time; PT:A, Preschool Trails: A response time; WJ VM, Woodcock-Johnson III Visual Matching subtest score; Lang, Woodcock-Johnson III Verbal Comprehension subtest score.

Variable distributions were examined for skewness and kurtosis prior to analysis, with outliers trimmed to within 3 *SD* of the mean. Response times also were log transformed, given evidence for significant skew. All models were constructed in MPLUS version 7.11 (Muthen and Muthen, 2012). Figure 1 describes the model of EC and processing speed, which initially was examined at each individual study age point. As shown, the Visual Matching subtest score was used as a statistical “anchor” for the processing speed factor, as it is a well-used, standardized measure of processing speed. Three other dependent measures from the baseline executive task conditions, namely mean response time for the Shape School baseline naming condition, mean response time for the Big-Little non-conflict trials, and Trails-P:A mean response times, were loaded onto this factor as processing speed measures. These response time variables were reverse-scaled in all presented models to enhance interpretability. In addition, all EC indicators were loaded onto the processing speed factor, thereby allowing any of the variance that EC conditions shared with the less complex, baseline processing conditions to be captured by the processing speed latent. To account for the variance in executive tasks that was shared with language, we used the only language assessment available in this study, the Verbal Comprehension subtest score from the BIA. Given that the Visual Matching and Verbal Comprehension subtests were administered only at study entry and exit, performance at the age point closest to executive task administration was used in these models. Using an approach similar to Lee et al. (2013), all residuals from the executive measures also were regressed on the Verbal Comprehension task, thus covarying for differences in language proficiency at the manifest level. Finally, executive task conditions were cross-loaded with an EC latent, which captured all of the residual shared variance between manifest executive tasks that was not accounted for by processing speed or the language covariate. All correlations between the latent factors and the latent factors and language proficiency were set to 0, as is common in multidimensional measurement models. Conceptually, this parameterization means that the model describes the contributions of the latent variables to manifest task performance if these variables are assumed to be orthogonal at the construct level. Where dependent measures had been extracted from the same task (e.g., Shape School baseline, Inhibit, and Switch conditions), their residuals were allowed to co-vary on the basis that (1) without accounting for their shared method variance, dependent variables extracted from the same task may have shown spuriously inflated loadings on the latent constructs and thereby clouded understanding of how the latent variables each contribute to task performance, and (2) initial analyses suggested significant improvement in model fit when these residuals were allowed to correlate.

After performing this descriptive analysis of the EC—processing speed overlap in each age group, we extended the analysis to more formally assess statistical changes in the strength of the EC factor loadings over time. This involved combining the models for each age point into a single model and then iteratively constraining the factor loadings to be equal at all age points. Where equality constraints caused a reduction in overall model fit, as evaluated with a chi-squared difference test (Kline, 2011), the loading was freed at one or more age points and the model was re-evaluated.

**Figure 2:**
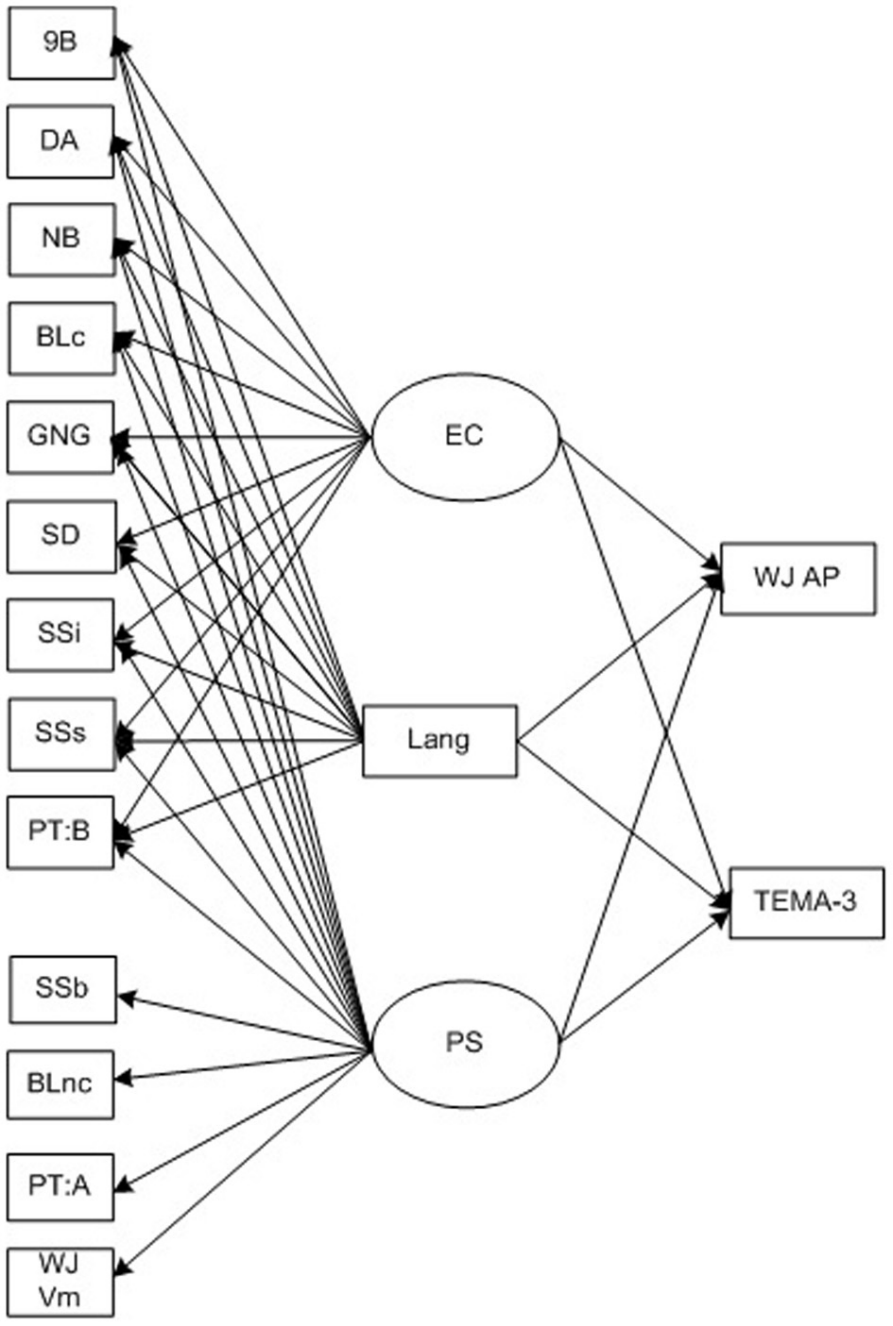
**Model of executive control, processing speed and language achievement as predictors of mathematics achievement over the preschool period**. EC, Executive Control; PS, Processing Speed; 9B, Nine Boxes maximum correct run; DA, Delayed Alternation score; NB, Nebraska Barnyard trials correct; BLc, Big-Little conflict trial accuracy; SD, Snack Delay movement score; SSi, Shape School Inhibit Accuracy; SSs, Shape School Switch accuracy; PT:B, Preschool Trails: B efficiency; SSb, Shape School Baseline naming response time; BLnc, Big-Little non-conflict trail response time; PT:A, Preschool Trails: A response time; WJ VM, Woodcock-Johnson III Visual Matching subtest; Lang, Woodcock-Johnson III Verbal Comprehension subtest score; WJ AP, Woodcock-Johnson III Applied Problems subtest score.

In the final stage of analysis, we examined the relation of EC and processing speed at each age to mathematics performance. As shown in Figure 2, for each independent age point, TEMA-3 and WJ-III Applied Problem subtest scores from the same time point and then every successive assessment point were regressed on processing speed, EC and on the language covariate.

## Results

### Descriptive profile of performance on executive tasks across the preschool period

Table 1 presents descriptive statistics for executive, processing speed and language proficiency measures, as well as the correlations between these tasks at different age points. For Shape School baseline, Big-Little non-conflict and Trails-P:A response times, higher scores reflect slower speed and, therefore, worse performance, whereas scores for all other tasks are positively scaled. Note that in many cases, correlations between executive and processing speed measures were as robust as correlations among the executive tasks themselves, highlighting the interrelations among these putative dimensions of cognition. Children's accuracy on the executive task conditions increased dramatically with age, as did the speed of their responses on non-executive task conditions. A multivariate ANOVA with age group as a predictor supported this pattern of improvement with age, with all univariate effects also significant, *F*_(36, 2869.7)_ = 38.54, Wilk's λ_(36)_ = 0.31, *p* < 0.001. Independent of the multivariate effect of age, there was no significant overall effect of study entry cohort, suggesting little impact of repeated testing on children's executive task performance, *F*_(36, 2869.7)_ = 1.29, Wilk's λ_(36)_ = 0.95, *p* = 0.11.

**Table 1:** **Descriptive statistics and correlations between executive control, processing speed, and language measures across the preschool period**.

**Measure**	***M* (*SD*)**	**1**	**2**	**3**	**4**	**5**	**6**	**7**	**8**	**9**	**10**	**11**	**12**	**13**
**AGE 3 YEARS (*n* = 228)**														
Nine boxes maximum correct run	4.32 (1.60)													
Delayed alternation summary score	−0.37 (3.96)	0.09												
Nebraska barnyard summary score	2.88 (1.57)	0.13^*^	0.19^**^											
Big little % correct conflict trials	0.29 (0.29)	0.08	0.23^***^	0.30^***^										
Go/No-Go *d*'	0.26 (0.75)	0.03	0.09	0.14^*^	0.18^**^									
Snack delay epochs with no movement	12.21 (11.42)	0.07	0.04	0.26^***^	0.24^***^	0.23^***^								
Shape school inhibit % correct	0.38 (0.42)	−0.02	0.18^*^	0.27^***^	0.36^***^	0.28^***^	0.26^***^							
Shape school switch % correct	0.26 (0.26)	0.03	0.15^*^	0.58^***^	0.25^**^	0.15	0.17^*^	0.50^***^						
Preschool trails B efficiency	0.70 (0.19)	0.02	0.07	0.33^***^	0.20^**^	0.15^*^	0.31^***^	0.27^***^	0.28^***^					
Big little non-conflict response time	3.21 (8.12)	−0.07	−0.05	−0.17^*^	−0.01	−0.09	−0.16^*^	−0.12	−0.08	−0.06				
Shape school A response time	2.30 (2.09)	0.00	−0.12	−0.34^***^	−0.23^**^	−0.23^**^	−0.24^***^	−0.14	−0.19^*^	−0.16^*^	0.24^***^			
Preschool trails A response time	7.51 (6.18)	−0.14^*^	−0.18^**^	−0.24^***^	−0.17^*^	−0.10	−0.04	−0.14	−0.17^*^	0.22^**^	−0.02	0.24^***^		
Visual matching study entry	413.45 (10.70)	0.07	0.10	0.41^***^	0.30^***^	0.16^*^	0.33^***^	0.24^***^	0.28^***^	0.19^*^	−0.16^*^	−0.32^***^	−0.22^**^	
Verbal comprehension study entry	436.60 (6.92)	0.05	0.17^**^	0.46^***^	0.23^***^	0.15^*^	0.26^***^	0.37^***^	0.40^***^	0.22^**^	−0.11	−0.22^**^	−0.26^***^	0.31^***^
**AGE 3.75 YEARS (***n*** = 276)**														
Nine boxes maximum correct run	4.92 (1.71)													
Delayed alternation summary score	2.61 (4.85)	0.09												
Nebraska barnyard summary score	4.90 (2.07)	0.14^*^	0.19^**^											
Big little % correct conflict trials	0.64 (0.34)	0.08	0.22^***^	0.43^***^										
Go/No-Go *d*'	1.38 (1.01)	0.11	0.11	0.25^***^	0.27^***^									
Snack delay epochs with no movement	17.96 (10.93)	0.08	0.10	0.20^***^	0.24^***^	0.30^***^								
Shape school inhibit % correct	0.75 (0.37)	0.05	0.12^*^	0.33^***^	0.36^***^	0.42^***^	0.25^***^							
Shape school switch % correct	0.57 (0.29)	0.14^*^	0.17^**^	0.35^***^	0.51^***^	0.33^***^	0.22^***^	0.48^***^						
Preschool trails B efficiency	0.82 (0.13)	0.04	0.07	0.18^**^	0.13^*^	0.09	0.10	0.28	0.20					
Big little non-conflict response time	1.59 (0.83)	−0.04	−0.03	−0.16^**^	0.11	−0.21^***^	−0.11	−0.13^*^	−0.10	−0.05				
Shape school A response time	1.26 (0.60)	−0.06	−0.13	−0.26^***^	−0.18^**^	−0.31^***^	−0.16^**^	−0.33^***^	−0.22^***^	−0.02	0.30^***^			
Preschool trails A response time	4.34 (3.52)	−0.10	−0.12	−0.36^***^	−0.27^***^	−0.31^***^	−0.28^***^	−0.16	−0.21^***^	0.04	0.08	0.19^**^		
Visual matching study entry	416.90 (13.28)	0.02	0.18^**^	0.28^***^	0.23^***^	0.23^***^	0.13^*^	0.19^**^	0.37^***^	−0.08	−0.10	−0.20^**^	−0.21^**^	
Verbal comprehension study entry	438.13 (7.74)	0.09	0.06	0.32^***^	0.30^***^	0.26^***^	0.22^***^	0.31^***^	0.42^***^	0.06	−0.11	−0.20^***^	−0.34^***^	0.48^***^
**AGE 4.5 YEARS (*n* = 319)**														
Nine boxes maximum correct run	5.23 (1.79)													
Delayed alternation summary score	5.99 (5.51)	0.09												
Nebraska barnyard summary score	6.98 (2.60)	0.17^**^	0.23^***^											
Big little % correct conflict trials	0.85 (0.21)	0.06	0.14^*^	0.34^***^										
Go/No-Go *d*'	2.30 (0.82)	0.02	0.18^**^	0.25^***^	0.20^***^									
Snack delay epochs with no movement	21.85 (9.40)	0.16^**^	0.23^***^	0.29^***^	0.16^**^	0.31^***^								
Shape school inhibit % correct	0.92 (0.18)	0.00	0.12^*^	0.21^***^	0.16^**^	0.32^***^	0.23^***^							
Shape school switch % correct	0.73 (0.24)	0.07	0.18^**^	0.37^***^	0.40^***^	0.33^***^	0.30^***^	0.29^***^						
Preschool trails B efficiency	0.86 (0.12)	0.09	0.16^**^	0.19^***^	0.13^*^	0.14^*^	0.16^**^	0.16^**^	0.22^***^					
Big little non-conflict response time	1.28 (0.49)	−0.10	−0.20^***^	−0.20^***^	0.14^*^	−0.22^***^	−0.20^***^	−0.18^**^	−0.15^*^	0.01				
Shape school A response time	1.02 (0.38)	−0.07	−0.13^*^	−0.13^*^	−0.08	−0.22^***^	−0.20^***^	−0.07	−0.12^*^	−0.03	0.34^***^			
Preschool trails A response time	2.21 (1.81)	−0.09	−0.17^**^	−0.38^***^	−0.18^**^	−0.30^***^	−0.30^***^	−0.13^*^	−0.28^***^	0.10	0.35^***^	0.38^***^		
Visual matching study exit	454.77 (11.48)	0.07	0.18^**^	0.34^***^	0.30^***^	0.25^***^	0.22^***^	0.18^**^	0.24^***^	0.08	−0.15^*^	−0.26^***^	−0.38^***^	
Verbal comprehension study exit	463.23 (12.76)	0.10	0.12^*^	0.45^***^	0.27^***^	0.27^***^	0.20^***^	0.16^**^	0.34^***^	0.00	−0.27^***^	−0.18^**^	−0.37^***^	0.44^***^
**AGE 5.25 YEARS (*n* = 363)**														
Nine boxes maximum correct run	5.73 (1.87)													
Delayed alternation summary score	7.53 (5.81)	0.11^*^												
Nebraska barnyard summary score	8.91 (2.41)	0.17^**^	0.14											
Big little % correct conflict trials	0.95 (0.08)	0.15^**^	0.11	0.38^***^										
Go/No-Go *d*'	2.74 (0.49)	0.17^**^	0.24	0.28^***^	0.25^***^									
Snack delay epochs with no movement	25.51 (9.85)	0.23^***^	0.03	0.24^*^	0.21^***^	0.20^***^								
Shape school inhibit % correct	0.96 (0.10)	0.03	0.05	0.13^*^	0.17^***^	0.09	0.15^**^							
Shape school switch % correct	0.84 (0.17)	0.12^*^	0.07	0.28^***^	0.28^***^	0.23^***^	0.19^***^	0.23^***^						
Preschool trails B efficiency	0.90 (0.11)	0.09	0.13^*^	0.17^**^	0.19^***^	0.11^*^	0.12^*^	0.12^*^	0.22^***^					
Big little non-conflict response time	1.12 (0.33)	−0.10	−0.11^*^	−0.25^***^	−0.05	−0.19^***^	−0.17^**^	−0.06	−0.17^**^	−0.06				
Shape school A response time	0.88 (0.25)	−0.02	−0.06	−0.16^**^	0.04	−0.15^*^	−0.09	0.05	−0.12^*^	0.04	0.21^***^			
Preschool trails A response time	1.28 (0.68)	−0.07	−0.13^*^	−0.31^***^	−0.22^***^	−0.29^***^	−0.11^*^	−0.02	−0.03	0.10	0.19^***^	0.24^***^		
Visual matching study exit	456.18 (11.99)	0.06	0.05	0.34^***^	0.26^***^	0.27^***^	0.13^*^	0.05	0.30^***^	0.05	−0.15^**^	−0.23^***^	−0.33^***^	
Verbal comprehension study exit	464.28 (13.12)	0.03	−0.02	0.53^***^	0.34^***^	0.28^***^	0.14^*^	0.15^**^	0.27^***^	0.13^*^	−0.18^***^	−0.14^**^	−0.24^***^	0.45^***^

* p < 0.05;

** p < 0.01;

*** *p < 0.001*.

*Response times presented in seconds, where a longer response indicates slower speed. The Visual Matching score is a Rasch-based, positively scaled w score (Woodcock et al., 2001a)*.

### Structural relations between processing speed and executive control over the course of the preschool period

Table 2 presents a summary of the models for each separate study follow-up point. Specifically, for each separate age, the table shows the standardized coefficients for the model described in Figure 1, including the factor loadings for all dependent variables loaded on the processing speed and EC factors, as well as the regression coefficients for executive tasks regressed on the language proficiency covariate. At age 3 years, most executive tasks loaded significantly on processing speed (λ = 0.21−0.46, *p* < 0.05), the exception being Nine Boxes, λ = 0.11, *p* = 0. 16; Model χ^2^_(54)_ 61.68, *p* = 0.22; CFI = 0.98; RMSEA = 0.03. The majority of executive measures also showed significant relations with the language covariate, although these associations were higher for tasks with more verbal content (i.e., Shape School, Nebraska Barnyard). Very few measures loaded on the independent EC factor (λ = −0.08−0.23;*p* > 0.05). The exceptions were Nebraska Barnyard (λ = −0.58, *p* = 0.03) and the Shape School Switch condition (λ = −0.30, *p* = 0.01), which showed negative loadings. Despite the low loadings on the EC latent, a chi-squared difference test indicated that the model incorporating the EC factor was a significant improvement over a model where the EC loadings were set to 0, Δχ^2^ = 38.6 (9), *p* < 0.001, although this fit may have been driven by a particularly large increase in the explained variance for Nebraska Barnyard when the EC latent was included (*R*^2^Δ = 0.37).

**Table 2:** **Summary of standardized coefficients for multidimensional model by age group**.

	**λ (S.E) on processing speed factor**				**β (S.E) on Woodcock–Johnson III language comprehension**				**λ (S.E) on executive control**			
	**3**	**3.75**	**4.5**	**5.25**	**3**	**3.75**	**4.5**	**5.25**	**3**	**3.75**	**4.5**	**5.25**
Nine boxes maximum correct run	0.11	0.16	0.14	0.16^*^	0.03	0.06	0.06	0.01	−0.09	0.01	0.07	0.31^***^
	(0.08)	(0.08)	(0.08)	(0.07)	(0.07)	(0.06)	(0.06)	(0.06)	(0.10)	(0.09)	(0.08)	(0.07)
Delayed alternation correct-incorrect runs	0.21^**^	0.32^**^	0.28^***^	0.26^***^	0.15^***^	−0.01	0.03	−0.06	0.02	0.08	0.22^**^	0.20^*^
	(0.08)	(0.07)	(0.07)	(0.07)	(0.07)	(0.06)	(0.06)	(0.05)	(0.09)	(0.09)	(0.08)	(0.08)
Nebraska barnyard summary score	0.57^***^	0.52^***^	0.31^***^	0.32^***^	0.40^***^	0.21^***^	0.39^***^	0.48^***^	−0.58^*^	0.18^**^	0.32^***^	0.31^***^
	(0.08)	(0.07)	(0.07)	(0.06)	(0.06)	(0.06)	(0.05)	(0.04)	(0.27)	(0.11)	(0.07)	(0.07)
Big-Little conflict trials % correct	0.48^***^	0.51^***^	0.11	0.17^**^	0.18^***^	0.21^***^	0.31^***^	0.32^***^	0.12	0.25	0.39^***^	0.44^***^
	(0.07)	(0.12)	(0.08)	(0.07)	(0.07)	(0.06)	(0.06)	(0.05)	(0.12)	(0.19)	(0.07)	(0.07)
Go/No-Go *d'*	0.34^***^	0.40^***^	0.36^***^	0.39^***^	0.11	0.18^***^	0.31^***^	0.20^***^	0.18	0.30^**^	0.31^***^	0.26^**^
	(0.08)	(0.08)	(0.07)	(0.07)	(0.07)	(0.06)	(0.06)	(0.05)	(0.11)	(0.10)	(0.07)	(0.08)
Snack delay movement score	0.40^***^	0.31^***^	0.39^***^	0.19^**^	0.20^***^	0.15^*^	0.17^**^	0.08	−0.12	0.17	0.31^***^	0.41^***^
	(0.08)	(0.08)	(0.07)	(0.07)	(0.07)	(0.06)	(0.06)	(0.06)	(0.09)	(0.11)	(0.07)	(0.08)
Shape school condition B % correct	0.43^***^	0.21^***^	0.19^*^	0.03	0.34^***^	0.26^***^	0.11	0.16^**^	0.23	0.85^*^	0.37^***^	0.36^**^
	(0.08)	(0.12)	(0.08)	(0.08)	(0.07)	(0.06)	(0.06)	(0.06)	(0.15)	(0.38)	(0.10)	(0.07)
Shape school condition D % correct	0.42^***^	0.46^***^	0.26^**^	0.19	0.35^***^	0.35^***^	0.30^***^	0.25^***^	−0.30^*^	0.35	0.62^***^	0.37^***^
	(0.10)	(0.15)	(0.08)	(0.07)	(0.07)	(0.06)	(0.06)	(0.05)	(0.12)	(0.23)	(0.08)	(0.07)
Preschool trails B efficiency	0.32^***^	0.12^**^	0.07	0.04	0.29^***^	0.08	0.04	0.16^**^	−0.06	0.32	0.38^***^	0.37^***^
	(0.08)	(0.16)	(0.09)	(0.09)	(0.07)	(0.07)	(0.06)	(0.05)	(0.10)	(0.18)	(0.08)	(0.07)
Big little non-conflict Trial *M* response time	0.27^**^	0.31^***^	0.49^***^	0.37^***^								
	(0.08)	(0.08)	(0.06)	(0.07)								
Shape school condition A *M* response time	0.50^***^	0.47^***^	0.54^***^	0.39^***^								
	(0.07)	(0.08)	(0.06)	(0.07)								
Preschool trails A response time	0.24^***^	0.43^***^	0.70^***^	0.62^***^								
	(0.08)	(0.08)	(0.06)	(0.07)								
Woodcock-Johnson visual matching score	0.49^***^	0.25^**^	0.36^***^	0.37^***^								
	(0.07)	(0.07)	(0.07)	(0.07)								

* p < 0.05;

** p < 0.01;

*** *p < 0.001. All response times are reverse scored for interpretability*.

In the model for the 3.75 age group, the residual variance for Shape School Inhibit was negative, leading to a non-positive definite solution. Once the non-significant residual covariance between Shape School Inhibit accuracy and Shape School Switch accuracy was set to 0, the model converged and was positive definite, although the removal of this residual covariance results in a model that is not directly comparable to models for other age points. All measures loaded significantly on the processing speed latent, λ = 0.16−0.52, *p* < 0.05; Model χ^2^_(55)_ = 118.59, *p* < 0.001; CFI = 0.90; RMSEA = 0.06. Similarly, children's performance on most of the measures, with the exception of Nine Boxes, Delayed Alternation and Trails-P: B, was related to their language proficiency (β = 0.13−0.35, *p* < 0.05). After accounting for variance shared with processing speed and the language covariate, Nebraska Barnyard, Shape School switch and Go/No-Go loaded significantly and positively on EC. Although model fit statistics indicated that the model provided only an adequate fit to the data, it still provided significantly better fit than a model that did not incorporate an EC latent, Δχ^2^ = 24.80 (9), *p* = 0.003, with the increase in explained variance being greatest for the Go/No-Go task (*R*^2^Δ = 0.27).

At age 4.5 years, the majority of executive tasks cross-loaded on EC (λ = 0.22−0.62, *p* < 0.01; Model χ^2^_(54)_ = 122.8, *p* < 0.001; CFI = 0.91; RMSEA = 0.06), the exception being Nine Boxes. Most measures also loaded significantly on the processing speed factor. Nebraska Barnyard, Big-Little, Go/No-Go, Snack Delay, and the Shape School Switch condition also showed significant relations with the language covariate. The model including the latent EC factor was a significant improvement over a model where loadings on the EC factor were set to 0, Δχ^2^ = 41.34 (9), *p* < 0.001. The *R*^2^ values for the individual EC tasks also increased by 2–19% with the addition of the EC latent.

Finally, at age 5.25 years, all EC tasks showed significant loadings of similar magnitude on the EC latent (λ = 0.20−0.38, *p* < 0.05; Model χ^2^_(54)_ = 103.37, *p* < 0.001; CFI = 0.93; RMSEA = 0.05), although Shape School Inhibit, Shape School Switch and Trails-P: B no longer loaded significantly on processing speed. Again, the fit of this model was a significant improvement over a model where the loadings on the EC factor were set to 0, Δχ^2^ = 39.48 (9), *p* < 0.001^1^, the *R*^2^ values for the manifest variables increasing by 1–20%.

### Metric invariance of factor loadings over the course of the preschool period

Taken together, the above findings suggest a gradual, age-related increase in the strength and consistency of executive task loadings on the separate EC factor. To more formally evaluate whether these apparent changes in factor loadings were statistically significant, a longitudinal metric invariance analysis was conducted. A combined model, which included the EC and processing speed factors for all four age points, provided a poor fit to the data, even allowing for residual autocorrelations between measures administered at directly successive age points, χ^2^ = 1550.47 (1124), *p* < 0.001, CFI = 0.88, RMSEA = 0.03. The majority of factor loadings for EC at age 3 also could not be set equivalent with loadings at the later age points without a significant reduction in model fit. Exceptions were Shape School switch accuracy and Snack Delay, which could be set equivalent between ages 3 and 3.75. By age 3.75, most of the loadings of executive tasks on EC could be constrained equal to those at ages 4.5 and 5.25 years, although Snack Delay and Go/No-Go could not. Finally, the only task loading for EC that could not be constrained to equality between ages 4.5 and 5.25 years was Big- Little, overall Δχ^2^ = 45.88 (39), p = 0.09. Correlations between the EC factors also increased from β = 0.34 to.45 for EC at age 3 with EC at later ages to β = 0.84 for EC at age 4.5 with EC at 5.25 years, all *p*'s < 0.001, suggesting increasing stability in the distinct EC factor over time.

### Relations of processing speed, language and executive control to mathematics achievement

Table 3 shows the relations of EC, processing speed and the language proficiency covariate to mathematics achievement both at simultaneous and follow-up age points (see Figure 2 for a description of the model tested independently for each assessment point). Processing speed and language proficiency were robustly correlated with TEMA-3 and WJ-III Applied Problems performance at all time points. However, latent EC at 3 years did not predict mathematics achievement. In contrast, higher latent EC at 3.75 years was associated with higher concurrent TEMA-3 and Applied Problems performance, independent of processing speed and language proficiency, χ^2^_(76)_ = 149.24, *p* < 0.001; CFI = 0.93; RMSEA = 0.06. Similarly, EC at 3.75 was associated with higher TEMA-3 and Applied Problems performance at age 4.5 years, χ^2^_(76)_ = 151.07, p < 0.001; CFI = 0.93; RMSEA = 0.06. Higher latent EC at 4.5 years also was associated with higher Applied Problems performance both concurrently (Model χ^2^_(76)_ = 184.70, *p* < 0.001; CFI = 0.91; RMSEA = 0.07) and at the 5.25 year follow-up, Model χ^2^_(76)_ = 181.36, *p* < 0.001; CFI = 0.92; RMSEA = 0.07. Finally, EC at age 5.25 was independently related to TEMA-3 and Applied Problems performance at the same 5.25 year age point, Model χ^2^_(76)_ = 153.88, *p* < 0.001; CFI = 0.94; RMSEA = 0.05. Findings for all models were similar when the effect of study entry cohort was considered.

**Table 3:** **Summary of relations of processing speed, language, and executive control to mathematics outcomes across the preschool period**.

	**β (S.E) on processing speed factor**				**β (S.E) on Woodcock–Johnson III language comprehension**				**β (S.E) on executive control**			
	**3**	**3.75**	**4.5**	**5.25**	**3**	**3.75**	**4.5**	**5.25**	**3**	**3.75**	**4.5**	**5.25**
**TEMA-3 STANDARD SCORE**												
3 Years	0.48^***^				0.44^***^				−0.12			
	(0.06)				(0.06)				(0.09)			
3.75 Years	0.47^***^	0.71^***^			0.51^***^	0.30^***^			−0.16	0.18^*^		
	(0.07)	(0.05)			(0.05)	(0.05)			(0.10)	(0.08)		
4.5 Years	0.45^***^	0.59^***^	0.60^***^		0.40^***^	0.32^***^	0.43^***^		−0.19	0.23^*^	0.14	
	(0.07)	(0.07)	(0.07)		(0.06)	(0.06)	(0.05)		(0.10)	(0.09)	(0.11)	
5.25 Years	0.35^***^	0.61^***^	0.46^***^	0.47^***^	0.38^***^	0.24^***^	0.46^***^	0.48^***^	−0.11	0.15	0.19^*^	0.37
	(0.07)	(0.06)	(0.06)	(0.06)	(0.06)	(0.06)	(0.06)	(0.04)	(0.10)	(0.10)	(0.08)	(0.06)
**WOODCOCK-JOHNSON APPLIED PROBLEMS SCORE**												
3.75 Years	0.34^***^	0.50^***^			0.60^***^	0.45^***^			−0.06	0.31		
	(0.07)	(0.07)			(0.05)	(0.06)			(0.09)	(0.08)		
4.5 Years	0.43^***^	0.52^***^	0.51^***^		0.45^***^	0.38^***^	0.46^***^		−0.03	0.40	0.35	
	(0.06)	(0.08)	(0.08)		(0.06)	(0.06)	(0.05)		(0.10)	(0.08)	(0.10)	
5.25 Years	0.35^***^	0.59^***^	0.47^***^	0.48^***^	0.39^***^	0.31^***^	0.47^***^	0.50^***^	−00.09	0.21^*^	0.30	0.27
	(0.07)	(0.06)	(0.06)	(0.06)	(0.06)	(0.06)	(0.05)	(0.04)	(0.11)	(0.10)	(0.07)	(0.07)

* p < 0.05;

*** *p < 0.001*.

*Separate models were constructed for each EC assessment and mathematics outcome age*.

## Discussion

The marked overlap in adult performance on measures of EC and general processing speed has triggered debate regarding the validity of EC as a distinct, independent dimension of cognition (Rabitt, 1997; Salthouse, 2005). In early childhood, a period when both EC and processing speed improve dramatically, such issues related to the construct validity of EC intersect with questions regarding the nature of EC development and the potentially cascading impact of advancements in basic processing fluency on higher-order cognition. The aims of this study were to examine the overlap between measures of EC and processing speed at different preschool age points and test the predictive utility of EC in relation to children's mathematics achievement after accounting for the processing demands of early executive tasks. Findings indicate that EC and processing speed are highly intertwined in early childhood to the extent that their impact on executive task performance at age 3 years could not be cleanly parsed. As children age through the preschool period, EC progressively differentiates from processing speed, becomes more stable, and shows independent predictive relations with mathematics achievement. Not only does this study shed some light on the psychometric characteristics of early EC tasks, but it also provides insight into the developmental mechanisms that might facilitate executive proficiency in early childhood.

Cascade models posit that increases in processing speed facilitate the development of higher-order executive skills (e.g., Fry and Hale, 1996). Support for this hypothesis derives from studies showing that processing speed mediates the relation of age to EC. Yet mediation analyses cannot reveal potential changes in the interplay of processing speed and EC over development. Consistent with the cascade model, this study does suggest that processing speed contributes substantially to children's performance on executive tasks. Nonetheless, the study also provides evidence that there are qualitative shifts in the interface of these processes over time. All of the EC factor loadings at age 3 were negative or non-significant, indicating that a general processing speed factor is able to explain all of the overlap in children's performance and that any residual variance after the processing and language demands of executive tasks are accounted for is largely specific to individual tasks. It is possible that at this young age, children draw to a greater extent on baseline processing and language skills to perform executive tasks, meaning that variability in executive performance is driven primarily by individual differences in these skills. A second possibility is that measures are not sufficiently sensitive to distinct dimensions of cognition because of the high level of within-person variability in young children's motivation and fatigue, implying that the processing speed factor reflects a broader, non-specific characteristic, such as task engagement or attention. A final possibility is that processing speed and EC are too tightly intertwined and co-dependent in this young age group. Even basic processing of shapes and colors may to some extent involve effortful cognitive control because children have not yet mastered these concepts, making it difficult to disentangle the unique roles of EC and processing speed in behavioral performance.

The relations between executive tasks increased over time, with some tasks beginning to load positively on a separate EC factor by age 3.75, although tasks also continued to load consistently on the processing speed factor through the preschool period. Quicker information processing may provide a platform for EC by freeing up higher-order resources, enabling children to hold more rules or situational requirements in working memory. Processing speed may also facilitate inhibition of motor or vocal responses because activation of inhibitory control networks can occur more quickly. This tight coupling between general processing speed and EC may help to explain why deficits in executive task performance characterize so many psychological disorders and why childhood traumatic brain injury to any area of the brain is associated with lower EC task performance (Jacobs et al., 2011). Disruption to cortical circuitry, regardless of its area in the brain, is likely to slow neural processing and transmission, with consequent bottom-up effects on EC. Even in older children, processing speed appears to mediate a substantial part, although not all, of the relation between age and complex working memory task performance (Bayliss et al., 2005; Fry and Hale, 1996). Recent studies also suggest that slow processing speed explains much of the deficit in working memory and inhibitory control performance in children with ADHD relative to their typically-developing peers (Lijffijt et al., 2005; Karulunas and Huang-Pollack, 2013).

At all age points, language proficiency also predicted residual variance in executive performance that was not explained by processing speed. The strong links between language abilities and EC often are framed in terms of social interactions and cultural tools, which theoretically create a symbol system that children can use to represent concepts or rules or to engage in internalized speech that allows them to self-regulate (Vygotsky, 1978). Perhaps its unique relation to language through these symbolic codes serves in part to differentiate EC from more general processing speed.

By the end of the preschool period, the common requirements of task conditions that had been manipulated to capture EC clearly diverged from processing speed and formed a coherent latent construct that was relatively stable from age 4.5 to 5.25 years. From a psychometric perspective, these findings provide evidence for the divergent validity and sensitivity of executive measures from about age 4 years. The extraction of shared task variance above and beyond that associated with processing speed and language allows for greater confidence that cognition incorporates a distinct, top-down control system, which is engaged specifically when tasks include demands for cognitive flexibility, the on-line maintenance and updating of task-relevant information, or the inhibition of a prepotent response. It should also be noted that some measures appear to be stronger indicators of EC than others. Despite their strong basis in animal studies of prefrontal function, Nine Boxes and Delayed Alternation showed lower and somewhat inconsistent correlations with other measures. The combination of processing speed, EC, and language comprehension explained only a small proportion of the variance for these tasks (3–12%). In contrast, Nebraska Barnyard, Big-Little Stroop, Go/No-Go, and Snack Delay showed relatively consistent correlations with each other across the preschool age range, suggesting that they may be more reliable indicators of EC. Collectively, EC, processing speed and the language covariate explained 15–82% of the variance in children's performance on these measures at different ages, whereas the maximum amount of variance explained in studies where our group has modeled EC without accounting for overlap with processing speed at the manifest level is only 57% (Nelson et al., 2014; see Willoughby et al., 2013 for similar findings).

From a theoretical perspective, the age-related divergence of EC and processing speed supports the differentiation hypothesis, where cognitive systems are thought to become progressively specialized over time (Hülür et al., 2011). Functional MRI studies show that, as children's performance on EC tasks improves, neural activation patterns become more focal and localized to regions of the brain that are typically activated when adults perform EC tasks (Durston et al., 2006; Rubia et al., 2006). Bell and Wolfe (2007) found that, in infancy, EEG activity during a working memory task was diffuse across the scalp. In the same group of children at age 4.5 years, however, EEG activity during working memory tasks was localized coherently at frontal electrode sites. There also is increasing development of long-range neural connections across childhood that presumably allow disparate neural systems to communicate more effectively (Fair et al., 2007). The gradual fractionation of EC abilities from processing speed evident in the current study is in line with this movement from a more diffuse activation of neural networks to the functional specialization of cortical circuits that coordinate cognitive control. However, it is also important to note that although EC appeared gradually to differentiate from processing speed, separate inhibition and working memory components of EC were not evident even by the final time point of this preschool study.

Processing speed and language proficiency were strong predictors of children's mathematics performance across the preschool years, whereas latent EC at age 3 years was not related to mathematics achievement once the processing speed and language demands of the EC tasks had been accounted for. Note that we are not suggesting that executive task performance at age 3 years is not a useful predictor of later mathematics achievement. As described in our earlier work, children's performance on many of the EC tasks at age 3 years correlates moderately with their mathematics achievement through the preschool period (Clark et al., 2013). What is clear is that the distinctions between EC and processing speed are not as clear-cut at age 3 and children's general processing speed may in fact drive the correlation between executive task performance and later mathematics. From age 3.75 years, EC did show independent correlations with mathematics achievement over and above individual differences in basic processing abilities and language. These findings provide more support for the construct validity and utility of EC, at least as assessed later in the preschool period. They also provide compelling evidence for the importance of both general processing speed and EC in children's early mathematics acquisition. Processing speed may reflect a central limiting mechanism that constrains or enhances children's ability to quickly retrieve or activate representations such as shapes, words or digits that are essential for mathematics. However, EC likely plays an added role in allowing for the maintenance and manipulation of these representations, which is essential for on-line mathematics problem solving. It will be important to extend these models to older age groups. Conceivably, the role of EC in mathematics could continue to increase over time. However, it is also possible that increasingly automatic and fluent numeric processing might eventually dampen the requirements for EC as children learn, resulting in differential relations to components of mathematics that have been mastered and those that are not as fluent over time.

It is important to note some limitations of the study. First, it is difficult to obtain pure measures of processing speed and measures of general reaction time may reflect other aspects of performance, including speed-accuracy trade-offs or lapses in attention (Schmiedek et al., 2007). The use of a factor score capturing variance from very different types of tasks was helpful in addressing this issue. Second, while it would have been ideal to construct a factor for language proficiency, constraints on the number of assessments that young children can feasibly complete limited our ability to acquire multiple indicators of language. Finally, in a recent study of EC in school-aged children, reaction times for baseline and executive task conditions could not be separated into distinct factors, whereas accuracy measures did form distinct EC components, highlighting an important influence of the type of indicator chosen on the measurement model for EC (van der Ven et al., 2013). As in most studies of preschoolers, we used accuracy or efficiency measures for the executive conditions of the EC tasks. While unlikely, given the use of varied scoring methods across tasks, is possible that the distinction between EC and processing speed in the later age groups is an artifact of the fact that most of the processing speed indicators were reaction times and most EC indicators were accuracy/efficiency measures.

Despite these limitations, this study clearly adds to the understanding of the nature and importance of EC by demonstrating dynamic changes in the overlap between processing speed and EC in early childhood and a qualitative re-organization of these interfacing processes over time. Early in the preschool period, executive tasks may not be sensitive indicators of an independent EC construct because EC is so intertwined with children's fluency of information processing. As children mature and their processing speed improves, a distinct EC construct plays a greater role in their EC task performance and this EC factor relates independently to children's developing mathematics proficiency. A key message from the study is that there is cause for optimism regarding the potential of specific EC assessment and intervention to address some of the pervasive discrepancies in children's academic readiness. This enthusiasm should be tempered, however, with the recognition of a corpus of psychological research demonstrating that the basic fluency with which children process information is an underpinning platform for intellectual development.

### Conflict of interest statement

The authors declare that the research was conducted in the absence of any commercial or financial relationships that could be construed as a potential conflict of interest.

## Footnotes

^1^Given that an independent EC factor was evident at age 5.25, we proceeded to test a two-factor EC structure with separate working memory and inhibitory control factors. A model with Nine Boxes, Delayed Alternation, and Nebraska Barnyard loaded on a working memory factor and other tasks loaded on an Inhibitory control factor did not provide better fit to the data, Δχ^2^ = 0.26_(1)_, *p* = 0.61 and the correlation between factors was high (*r* = 0.91). Similarly, a model where Shape School Switch and Trails-P: B were loaded on the inhibitory control factor was not superior to the unitary model, Δχ^2^ = 0.003_(1)_, *p* = 0.96. In keeping with previous research in this age group, a unitary factor was the preferred model of EC, even when the processing speed and language requirements of executive tasks were accounted for.
